# Tenascin C Promotes Glioma Cell Malignant Behavior and Inhibits Chemosensitivity to Paclitaxel via Activation of the PI3K/AKT Signaling Pathway

**DOI:** 10.1007/s12031-021-01832-8

**Published:** 2021-04-19

**Authors:** Qingping Zhang, Binchu Xu, Fulan Hu, Xianjin Chen, Xinmin Liu, Qinghua Zhang, You Zuo

**Affiliations:** 1 Department of Neurosurgery, Huazhong University of Science and Technology Union Shenzhen Hospital, The 6th Affiliated Hospital of Shenzhen University Health Science Center (Shenzhen Nanshan People’s Hospital), Shenzhen 518056, Guangdong, China; 2 Department of Neurosurgery, The Seventh Affiliated Hospital of Sun Yat-Sen University, Shenzhen, Guangdong China; 3 Department of Biostatistics and Epidemiology, School of Public Health, Shenzhen University Health Science Center, Shenzhen 518060, Guangdong, China; 4 Department of Neurosurgery, Southern University of Science and Technology Yantian Hospital, Shenzhen 518081, Guangdong, People’s Republic of China

**Keywords:** Glioma, Tenascin C, Cell function, Chemosensitivity, PI3K/AKT signaling

## Abstract

The present study aimed to detect the effect of tenascin C (TNC) on cell function and chemosensitivity to paclitaxel and phosphatidylinositol 3-kinase/protein kinase B (PI3K/AKT) signaling in glioma cells.

Human glioma cells U87, LN-229, T98G and U251 and normal human astrocytes were obtained, in which TNC expression was detected. The U87 cells and U251 cells were chosen and infected with lentivirus of control overexpression, TNC overexpression, control knockdown, and TNC knockdown for functional experiments. Rescue experiments were then performed to evaluate the effect of PI3K/AKT activator 740 Y-P on cell function and chemosensitivity to paclitaxel in TNC knockdown U251 cells. TNC mRNA and protein expression was elevated in glioma cells, including U87, LN-229, U251 and T98G cells, compared to normal human astrocytes. In U87 and U251 cells, TNC promoted proliferation while inhibiting apoptosis. In addition, TNC upregulated PI3K and p-AKT protein expression in U87 and U251 cells. As for chemosensitivity, TNC increased relative viability in U251 cells treated with 400 ng/mL and 800 ng/mL paclitaxel. In terms of stemness, TNC increased the sphere number per 1000 cells, CD44^+^CD133^+^ cell percentage and 1/stem cell frequency (assessed by extreme limiting dilution analysis) in U251 cells. In rescue experiments, 740 Y-P reduced the effect of TNC on proliferation, apoptosis, chemosensitivity to paclitaxel, and stemness in U251 cells.

TNC acts as an oncogenic factor by promoting cancer cell proliferation and stemness while inhibiting apoptosis and chemosensitivity to paclitaxel in glioma via modulation of PI3K/AKT signaling.

## Introduction

Tenascin C (TNC), a glycoprotein with a multi-modular structure that is mainly expressed in tendons and embryos, is a crucial regulator in embryonic development, predominantly via interaction with motile cells (Brosicke and Faissner 2015). The distribution of TNC in the human body is an intriguing topic of study. TNC is expressed at very low levels in adult tissues compared with that in embryos, although it is expressed most abundantly in stem cells, sites of inflammation or tissue trauma, and solid tumors in adults (Uenishi et al. 2014; Ning et al. 2019; Zhao et al. 2017; Leppanen et al. 2019). Based on its role in regulating cell adhesion by binding with fibronectin and in tissue regeneration, an increasing number of basic and clinical scientists have turned their attention to the potential value of TNC in oncology (Giuffrida et al. 2004; Sundquist et al. 2017). Since the first discovery regarding the role of TNC in cancers, over two decades have passed. Findings such as the involvement of TNC in tumor growth, metastasis and stemness have rejuvenated the research area of oncology (Sun et al. 2018; Sun et al. 2019; Yang et al. 2019).

Glioma is the most frequently diagnosed brain cancer; however, compared with other solid tumors, it presents with an extremely low prevalence of about six in every 100,000 persons, which still translates to a huge death rate worldwide (Dolecek et al. 2012). Originating from glial precursor cells, glioma is not only a carcinoma but also a brain disease; thus patients typically suffer from many neurological symptoms such as headache and seizure (Tanaka et al. 2013). Despite the various methods for treating glioma including resection, chemotherapy and radiation, and although the combination of chemotherapy and procarbazine has been found to prolong survival, the survival profiles of glioma patients remain one of the poorest (Buckner et al. 2014; Bell et al. 2020). A previous study reported that TNC overexpression correlates with a pediatric brainstem glioma phenotype and a more unsatisfying survival profile (Qi et al. 2019). A few studies have also revealed a correlation between TNC and glioma etiology; however, an understanding of the precise regulatory function and the underlying mechanism of TNC in glioma remains elusive.

Therefore, we aimed to determine the effect of TNC on cancer cell function, chemosensitivity to paclitaxel and PI3K/AKT signaling in glioma.

## Materials and Methods

### Cell Culture and Reagents

Human glioma cells U87, LN-229, T98G and U251 were purchased from the American Type Culture Collection (ATCC, USA) or the European Collection of Authenticated Cell Cultures (ECACC, UK), and cultured with the suppliers’ recommended medium. Normal human astrocytes, purchased from Lonza (Switzerland), were cultured in astrocyte culture medium (Cell Applications, USA). TRIzol™ reagent, the PrimeScript™ RT reagent kit and TB Green Realtime PCR Master Mix were obtained from Thermo Fisher Scientific, Inc. (USA) and Takara Biomedical Technology (Beijing) Co., Ltd. (China), respectively. RIPA lysis buffer was obtained from Merck Sigma-Aldrich (USA). The BCA protein assay kit was supplied by Beyotime Institute of Biotechnology (China). Precast gel (4–20%) was obtained from Willget Biotech Co., Ltd. (China). Nitrocellulose filter membrane was obtained from Merck Millipore (Darmstadt, Germany). Pierce™ ECL Plus Western Blotting Substrate was purchased from Thermo Fisher Scientific, Inc. (USA). The cell counting kit-8 and annexin V-FITC apoptosis detection kit were obtained from Dojindo Molecular Technologies, Inc. (Japan) and Merck Sigma-Aldrich, (USA), respectively. Paclitaxel and PI3K activator 740 Y-P were purchased from Sangon Biotech (Shanghai) Co., Ltd. (China) and Selleck Chemicals (USA). B-27™ Supplement, recombinant human epidermal growth factor (EGF), recombinant human basic fibroblast growth factor (bFGF) and Dulbecco's modified Eagle medium/Nutrient Mixture F-12 (DMEM/F12) were purchased from Thermo Fisher Scientific, Inc. (USA). PI3 kinase p110α rabbit polyclonal antibody (1:1000), AKT rabbit polyclonal antibody (1:1000) and phospho-Akt rabbit polyclonal antibody (1:1000) were purchased from Cell Signaling Technology, Inc. (CST, UK). Tenascin-C rabbit polyclonal antibody (1:1000), GAPDH rabbit monoclonal antibody (1:5000) and HRP-labeled goat anti-rabbit IgG(H+L) (1:3000) were purchased from Abcam plc (UK). PE-labeled CD133 antibody and APC-labeled CD44 antibody were purchased from Becton, Dickinson and Company (USA). Lentivirus for overexpression of TNC (OE-TNC) and overexpression-negative control (OE-NC), which were constructed with a pLV-CMVIE-RFP-T2A-Puro vector, enveloped in a pMD2.G vector and psPAX2 vector, were completed by Hanbio Biotechlogy Co., Ltd. (China). Lentivirus for knockdown TNC (KD-TNC) and knockdown negative control (KD-NC), which were constructed with the pLV-U6-RFP-T2A-luc vector, were enveloped with pMD2.G vector and psPAX2 vector by Hanbio Biotechlogy Co., Ltd. (China). Polybrene and puromycin were provided by Beijing Solarbio Science & Technology Co., Ltd. (China). TNC primers (forward: 5′ AATGAGATGCGGGTCACAGA 3′, reverse: 5′ GATGGCAAATACACGGATAAAGT 3′) and GAPDH primers (forward: 5′ GACCACAGTCCATGCCATCAC 3′, reverse: 5′ ACGCCTGCTTCACCACCTT 3′) were synthesized by Sangon Biotech (Shanghai) Co., Ltd. (China).

### Lentivirus Infection

U251 and U87 cells were infected with OE-NC, OE-TNC, KD-NC and KD-TNC lentivirus with multiplicity of infection (MOI) of 20 for 24 h in the presence of 6 μg/ml polybrene. After cells were cultured for another 48 h, they were collected for the detection of TNC expression by RT-qPCR and western blot. The cells were then cultured with 2 μg/ml puromycin to screen the stably infected cells for 7 days. The expression of PI3K, AKT and p-AKT was detected by western blot.

### Cell Proliferation and Apoptosis

The proliferation and apoptosis of stably infected U251 and U87 cells were evaluated with the cell counting kit-8 and annexin V-FITC apoptosis detection kit, respectively. To complete the detection, the instructions of the kits were strictly followed.

### Drug Chemosensitivity

The stably infected U251 cells were seeded in 96-well plates for 24 h, after which the cells were cultured with medium containing 0, 100, 200, 400, 800 and 1600 ng/ml paclitaxel for another 24 h. Cell viability was evaluated using the cell-counting kit-8, with cells cultured with 0 ng/ml paclitaxel set as 100%.

### Sphere Formation Assay and Extreme Limiting Dilution Analysis (ELDA)

After collection, 1000 stably infected U251 cells were cultured with sphere formation medium DMEM/F12 containing 2% B-27™ supplement, 20 ng/ml bFGF and 20 ng/ml EGF for 7 days, and the number of spheres with diameter > 50 μm was counted. For the ELDA, stably infected U251 cells with numbers of 1000, 100 and 10 were each plated for 24 wells and cultured with sphere formation medium for 7 days. The wells with at least one sphere with a diameter > 50 μm were counted, and the data were calculated with ELDA (http://bioinf.wehi.edu.au/software/elda/).

### CD44-Positive and CD133-Positive (CD44^+^CD133^+^) Cell Assessment

The stably infected U251 cells were collected and resuspended. Then the APC-labeled CD44 antibody (1:50) and PE-labeled CD133 antibody (1:50) were incubated with cells in the dark on ice for 30 min. Lastly, cells were detected with a flow cytometer (BD, USA), and the percentage of CD44^+^CD133^+^ cells was analyzed with FlowJo 7.6 (BD, USA).

### 740 Y-P Treatment

For the treatment of 740 Y-P, culture medium containing 10 μM 740 Y-P was prepared. Then the stably infected U251 cells were incubated with 740 Y-P, followed by PI3K, AKT and p-AKT expression detection, cell proliferation and apoptosis assessment, drug chemosensitivity evaluation, sphere formation assay and ELDA detection, and CD44^+^CD133^+^ cell evaluation using the methods described above.

### Reverse Transcription Quantitative Polymerase Chain Reaction (RT-qPCR)

The total RNA in cells was extracted with TRIzol™ reagent, after which reverse transcription and qPCR were performed using the PrimeScript™ RT reagent kit and TB Green Realtime PCR Master Mix. The expression of TNC (with GAPDH as internal reference) was calculated using the 2^−ΔΔCt^ method.

### Western Blot

Cells were lysed, and total protein was extracted with RIPA lysis buffer. Then the BCA protein assay kit was used to quantify the concentration of protein. After thermal denaturing, 20 μg protein was loaded in and separated with 4–20% precast gel. Then the protein was transferred to a nitrocellulose filter membrane, followed by incubation with primary antibodies. After incubation with secondary antibody, the protein band was visualized with Pierce™ ECL Plus Western Blotting Substrate.

### Statistical Analysis

Data analysis and graph plotting were carried out in GraphPad Prism 7.02 software (GraphPad Software Inc., USA). All data are presented as mean ± standard deviation. Comparisons among groups were determined by one-way analysis of variance (ANOVA) followed by Dunnett's multiple comparisons test or Tukey’s multiple comparisons test. Direct comparison between two groups was determined by the *t* test. *P* < 0.05 was considered as statistically significant. *P* < 0.05, < 0.01 and < 0.001 are expressed as *, ** and ***, respectively. Non-significant results are marked as NS.

## Results

### TNC was Upregulated in Human Glioma Cells

**Fig. 1:**
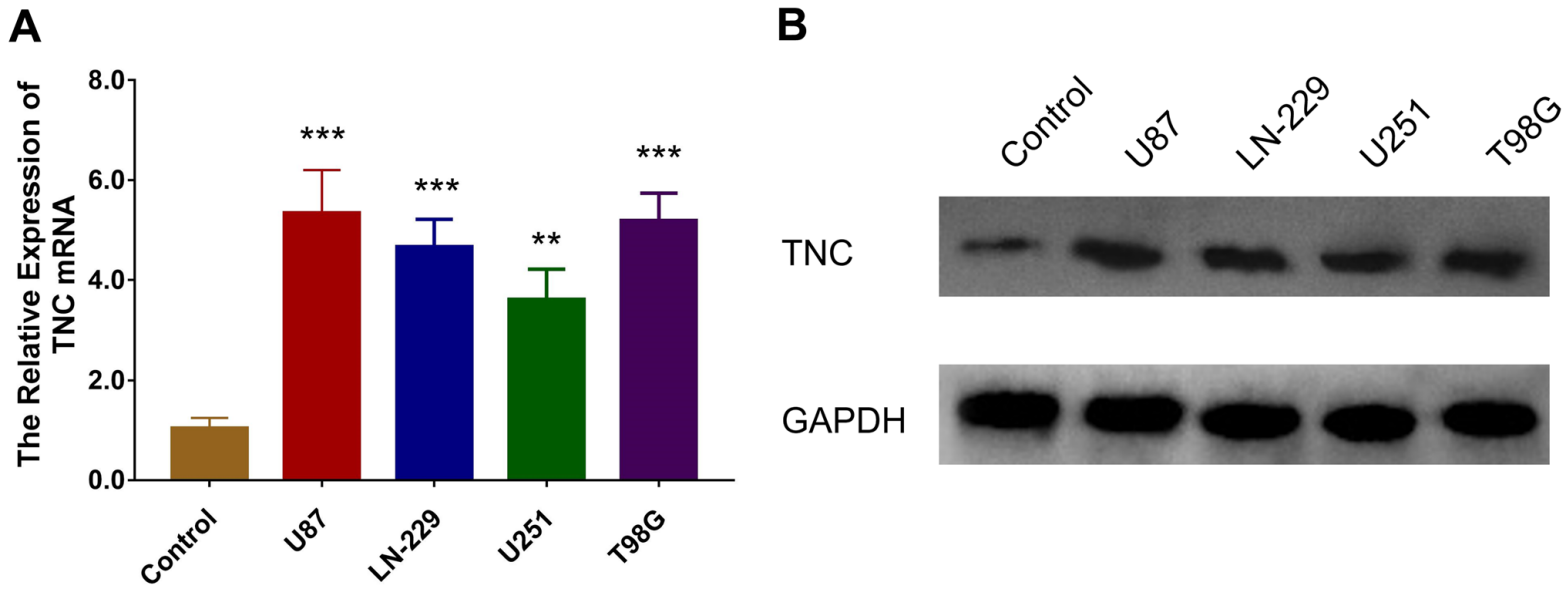
mRNA and protein expression of TNC in glioma cells. Comparison of TNC mRNA (**a**) and protein (**b**) expression between glioma cells, including U87, LN-229, U251 and T98G cells, and normal human astrocytes as control. *TNC* tenascin C; *GAPDH* glyceraldehyde-phosphate dehydrogenase

TNC mRNA was overexpressed in the human glioma cells, including U87 (*P* < 0.001), LN-229 (*P* < 0.001), U251 (*P* < 0.01) and T98G (*P* < 0.001) cells, compared to the normal human astrocytes (Fig. 1a), and TNC protein expression was also upregulated in U87, LN-229, U251 and T89G cells compared to normal human astrocytes (Fig. 1b). The U251 cell and U87 cell lines were then chosen for the subsequent experiments.

### TNC Regulated Human Glioma Cell Proliferation, Apoptosis and PI3K/AKT Signaling

**Fig. 2:**
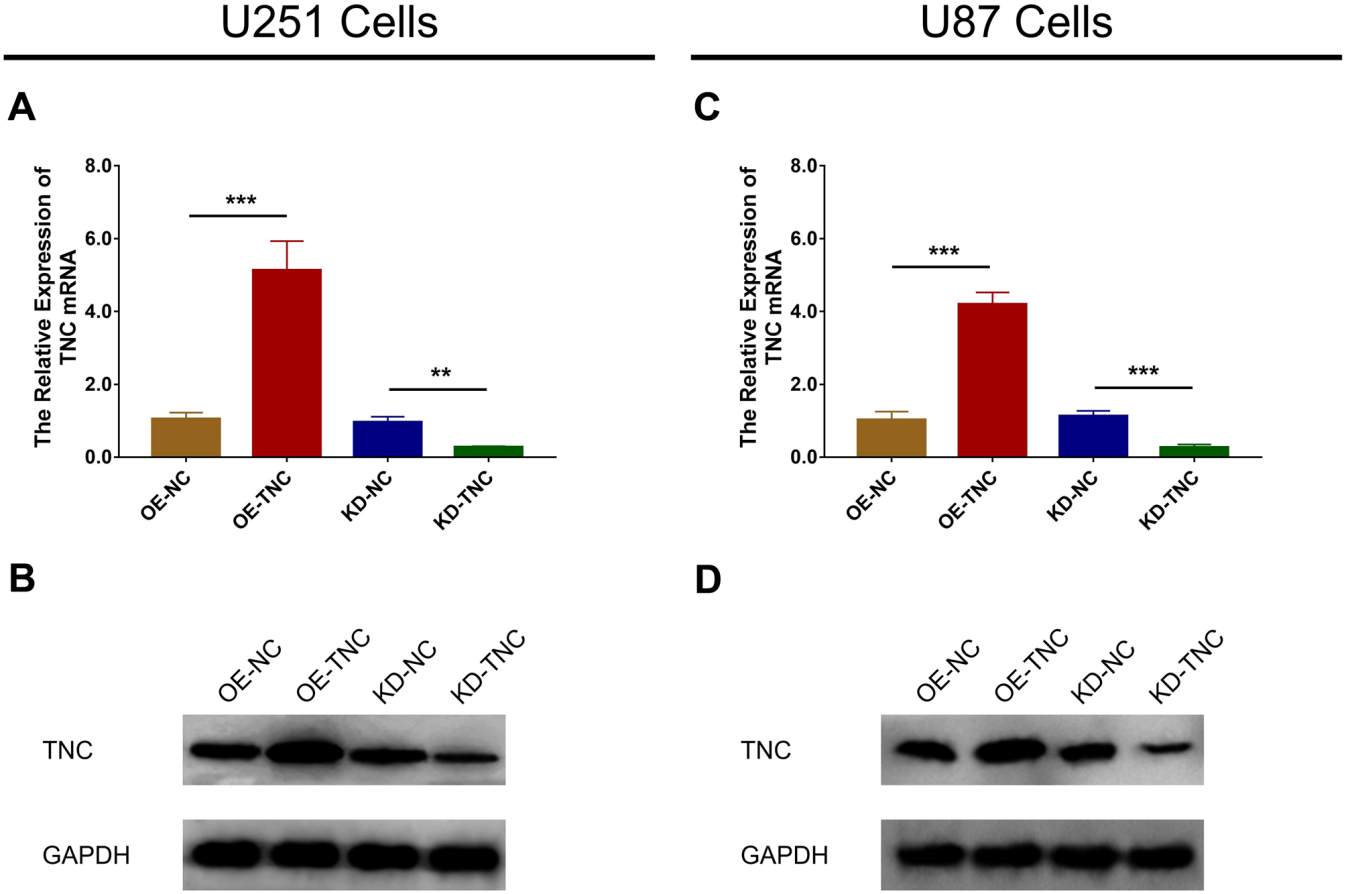
mRNA and protein expression of TNC in glioma cells after lentivirus infection. TNC mRNA (**a**) and protein (**b**) expression among the OE-NC, OE-TNC, KD-NC and KD-TNC groups in U251 cells. TNC mRNA (**c**) and protein (**d**) expression among the OE-NC, OE-TNC, KD-NC and KD-TNC groups in U87 cells. *TNC* tenascin C; *OE* overexpression; *NC* negative control; *KD* knockdown; G*APDH* glyceraldehyde-phosphate dehydrogenase

**Fig. 3:**
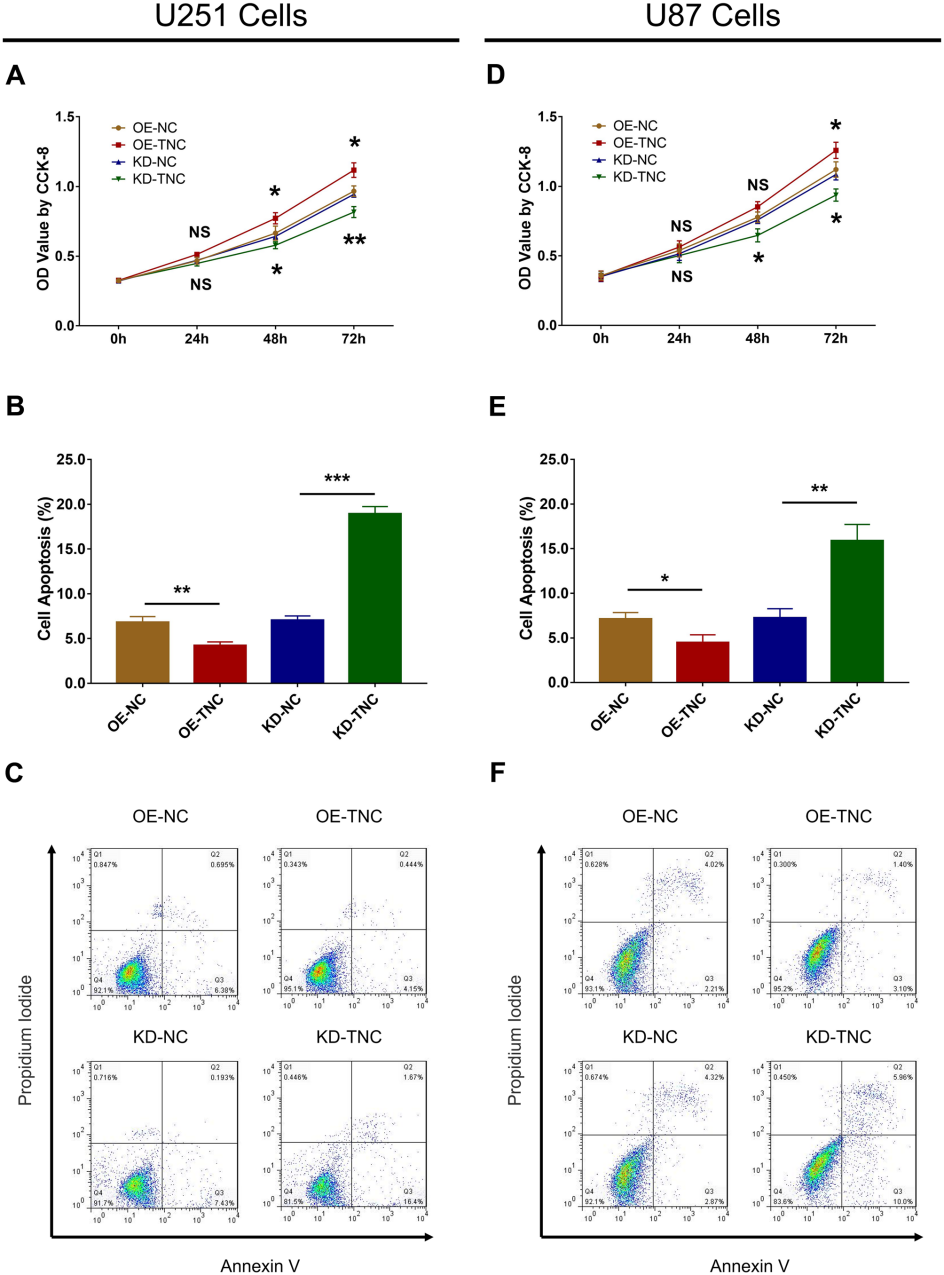
Cell proliferation and apoptosis regulated by TNC in glioma cells. The OD value (**a**) and cell apoptosis percentage (**b**, **c**) among the OE-NC, OE-TNC, KD-NC and KD-TNC groups in U251 cells. OD value (**d**) and cell apoptosis percentage (**e**, **f**) among the OE-NC, OE-TNC, KD-NC and KD-TNC groups in U87 cells. *TNC* tenascin C; *OD* optical density; *CCK-8* cell-counting kit-8; *OE* overexpression; *NC* negative control; *KD* knock down

**Fig. 4:**
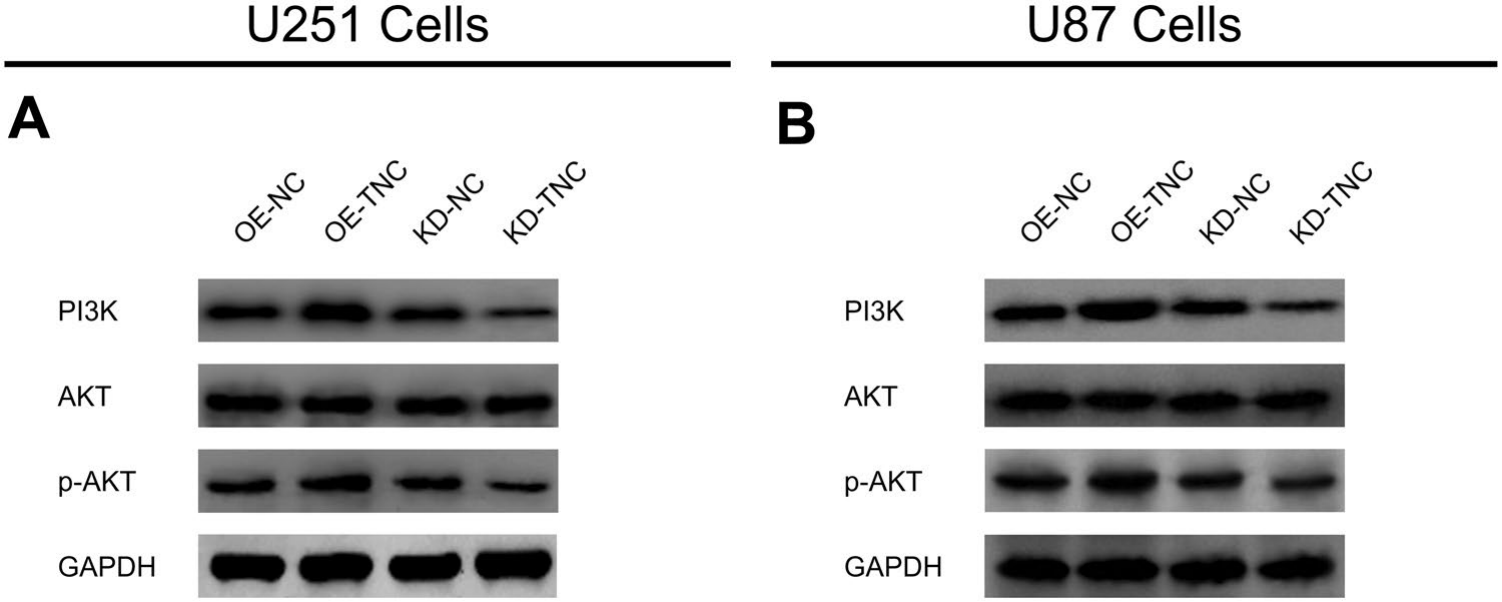
PI3K/AKT signaling regulated by TNC in glioma cells. PI3K, AKT and p-AKT protein expression among the OE-NC, OE-TNC, KD-NC and KD-TNC groups in U251 cells (**a**) and U87 cells (**b**). *PI3K* phosphatidylinositol 3-kinase; *AKT* protein kinase B; *p-AKT* phosphorylated-AKT; *TNC* tenascin C; *OE* overexpression; *NC* negative control; *KD* knockdown; *GAPDH* glyceraldehyde-phosphate dehydrogenase

Expression of TNC mRNA (Fig. 2a) and protein (Fig. 2b) was upregulated in the OE-TNC group compared to the OE-NC group (*P* < 0.001), while expression was downregulated in the KD-TNC group relative to the KD-NC group (*P* < 0.01) in U251 cells. Similarly, expression of TNC mRNA (*P* < 0.001) and protein (*P* < 0.001) was increased in the OE-TNC versus the OE-NC group (Fig. 2c) but was decreased in the KD-TNC group relative to the KD-NC group (Fig. 2d) in U87 cells. These results indicate that the transfection was successful. Further experiments revealed that TNC positively regulated cell proliferation at 48 h and 72 h post-transfection (Fig. 3a) but negatively modulated cell apoptosis in U251 cells (Fig. 3b, c) (all *P* < 0.05). Additionally, cell proliferation was enhanced at 72 h post-transfection (Fig. 3d) but cell apoptosis (Fig. 3e, f) was inhibited by TNC in the U87 cells (all *P* < 0.05). Moreover, PI3K and p-AKT protein expression was upregulated by TNC in both U251 cells (Fig. 4a) and U87 cells (Fig. 4b).

### TNC Regulated Chemosensitivity and Stemness in Human Glioma Cells

**Fig. 5:**
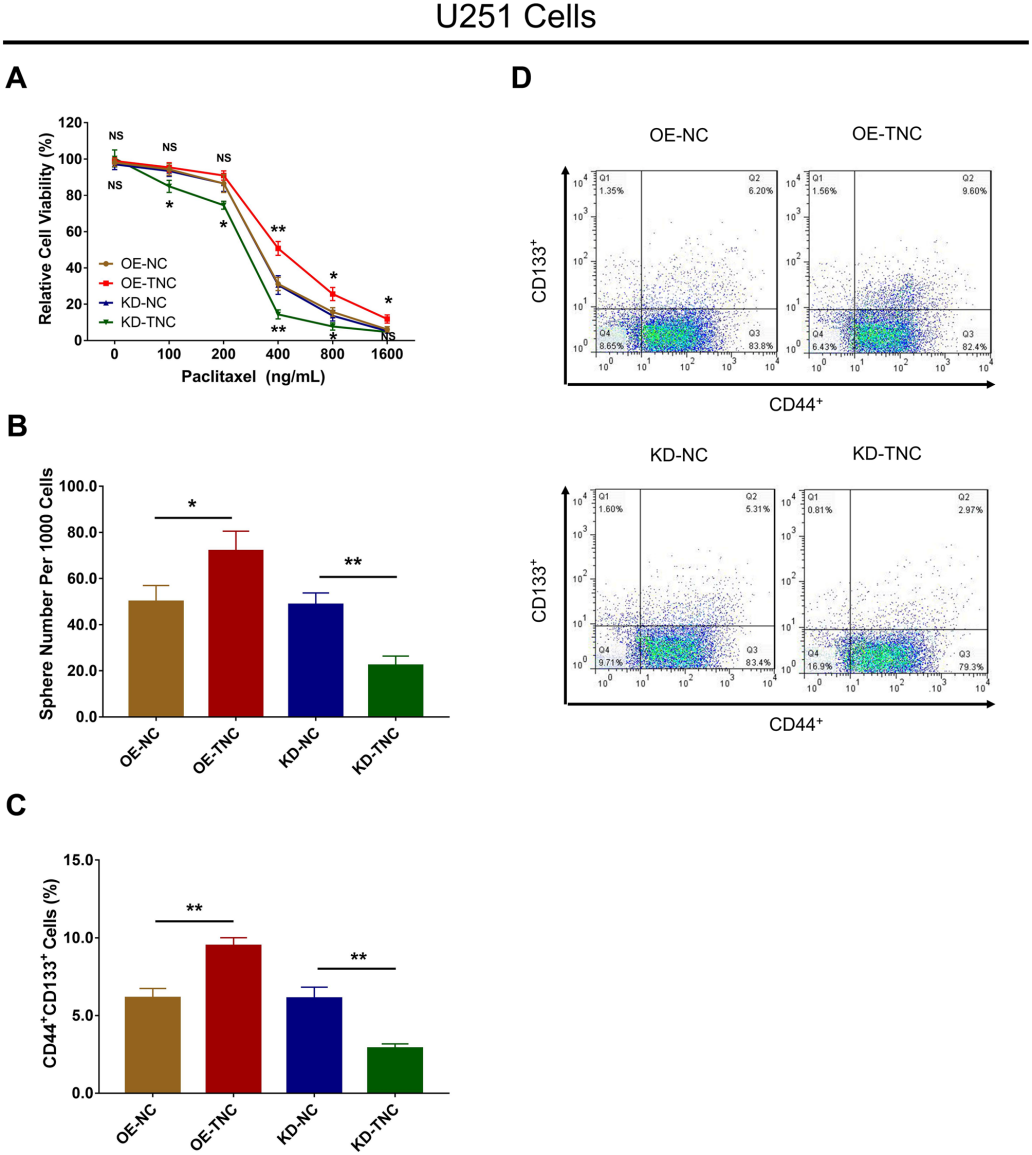
Chemosensitivity to paclitaxel and stemness regulated by TNC in glioma cells. Relative cell viability (**a**), sphere number per 1000 cells (**b**), and CD44^+^CD133^+^ cell percentage (**c**, **d**) among the OE-NC, OE-TNC, KD-NC and KD-TNC groups in U251 cells. *TNC* tenascin C; *OE* overexpression; *NC* negative control; *KD* knockdown

Subsequently, U251 cells were selected for further experiments, which revealed that TNC overexpression enhanced the relative viability in cells treated with 400 ng/mL, 800 ng/mL and 1600 ng/mL paclitaxel, while TNC knockdown repressed relative viability in cells treated with 100 ng/mL, 200 ng/mL, 400 ng/mL and 800 ng/mL paclitaxel (all *P* < 0.05) (Fig. 5a). As to cell stemness, TNC enhanced the sphere number per 1000 cells (Fig. 5b) and the CD44^+^CD133^+^ cell percentage in U251 cells (Fig. 5c, d) (all *P* < 0.05). With regard to ELDA data, TNC increased the 1/stem frequency in U251 cells (all *P* < 0.01) (Table 1).

**Table 1:** ELDA data for stably infected cells

Groups	Limiting dilution (wells)			1/Stem cell frequency			*P* value
	1000	100	10	Lower	Estimate	Upper	
OE-NC	23/24	18/24	8/24	159.3	98.5	61.0	0.00806
OE-TNC	24/24	19/24	10/24	71.4	45.8	29.5	
KD-NC	22/24	19/24	7/24	227.4	136.1	81.5	0.00673
KD-TNC	20/24	14/24	4/24	466.4	290.6	181.2	

*ELDA* extreme limiting dilution analysis

### PI3K/AKT Signaling Reversed the Effect of TNC Knockdown on Cell Function and Chemosensitivity in Human Glioma Cells

**Fig. 6:**
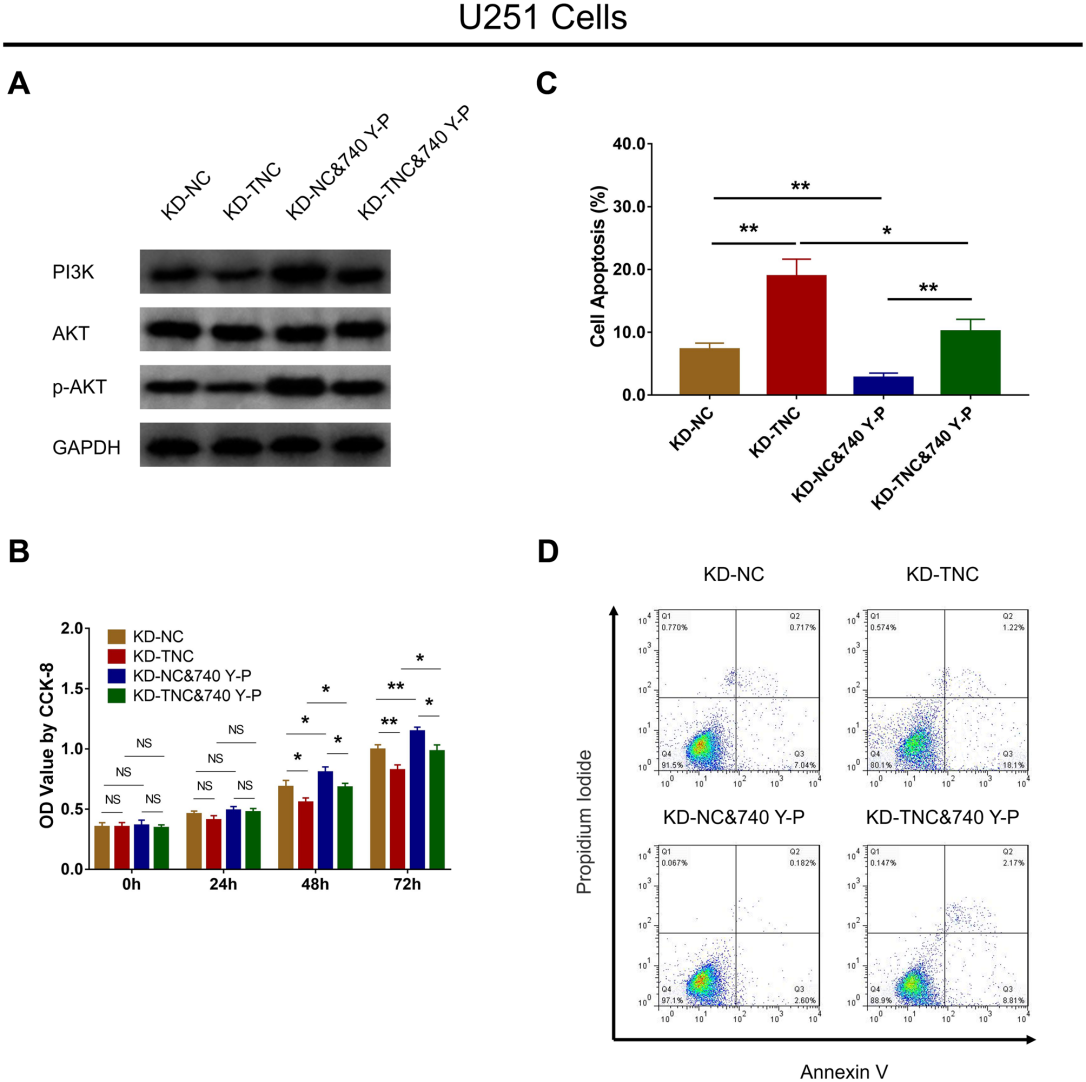
Effect of PI3K/AKT activator on proliferation and apoptosis in TNC knockdown glioma cells. PI3K, AKT and p-AKT protein expression (**a**), OD value (**b**) and cell apoptosis percentage (**c**, **d**) among the KD-NC, KD-TNC, KD-NC&740 Y-P and KD-TNC&740 Y-P groups in U251 cells. *PI3K* phosphatidylinositol 3-kinase; *AKT* protein kinase B; *TNC* tenascin C; *p-AKT* phosphorylated-AKT; *OD* optical density; *CCK-8* cell counting kit-8; *KD* knockdown; *NC* negative control; *GAPDH* glyceraldehyde-phosphate dehydrogenase

In U251 cells, TNC knockdown reduced PI3K and p-AKT protein expression, while treatment with 740 Y-P enhanced PI3K and p-AKT protein expression in KD-TNC cells (Fig. 6a). This suggests that the treatment with the PI3K/AKT activator 740 Y-P in U251 cells was successful. Afterward, TNC knockdown downregulated cell proliferation in U251 cells; however, 740 Y-P upregulated cell proliferation in KD-TNC U251 cells at 48 h and 72 h (all *P* < 0.05) (Fig. 6b). In terms of cell apoptosis, it was upregulated by TNC knockdown in U251 cells, but was downregulated by 740 Y-P in KD-TNC U251 cells (all *P* < 0.05) (Fig. 6c, d).

**Fig. 7:**
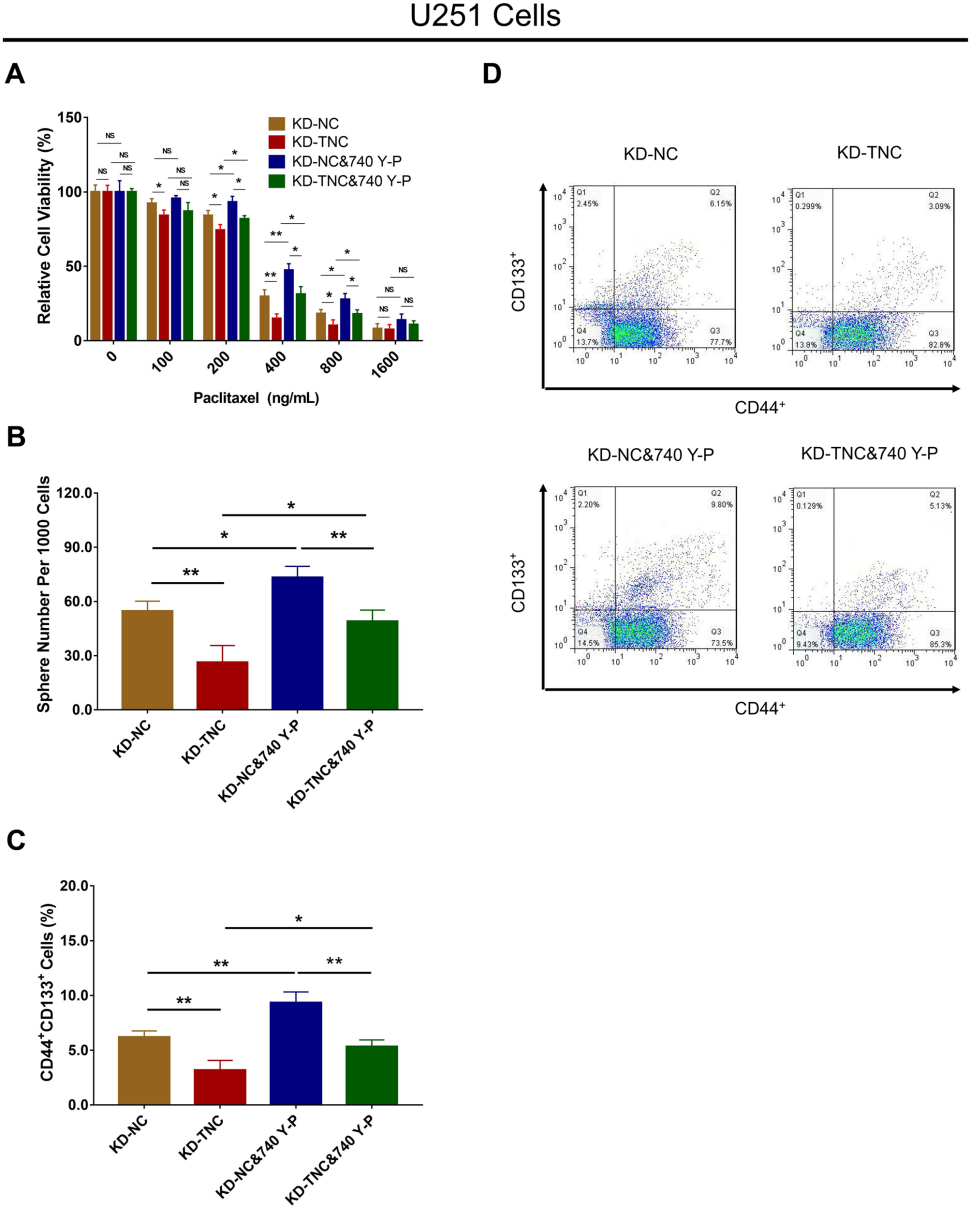
Effect of PI3K/AKT activator on chemosensitivity to paclitaxel and stemness in TNC knockdown glioma cells. U251 cell relative cell viability (**a**), sphere number per 1000 cells (**b**) and CD44^+^CD133^+^ cell percentage (**c**, **d**) among the KD-NC, KD-TNC, KD-NC&740 Y-P and KD-TNC&740 Y-P groups. *PI3K* phosphatidylinositol 3-kinase; *AKT* protein kinase B; *TNC* tenascin C; *KD* knockdown; *NC* negative control

With regard to chemosensitivity, TNC knockdown inhibited U251 relative cell viability; however, 740 Y-P promoted the relative viability of KD-TNC U251 cells under 200 ng/mL, 400 ng/mL and 800 ng/mL paclitaxel treatment (all *P* < 0.05) (Fig. 7a). As regards cell stemness, the sphere number per 1000 cells was decreased by TNC knockdown in U251 cells, but was increased by 740 Y-P in KD-TNC U251 cells (all *P* < 0.05) (Fig. 7b). In addition, TNC knockdown reduced CD44^+^CD133^+^ cell percentage in U251 cells, but 740 Y-P enhanced CD44^+^CD133^+^ cell percentage in KD-TNC U251 cells (all *P* < 0.05) (Fig. 7c, d). With regard to the ELDA data, TNC knockdown reduced 1/stem cell frequency in U251 cells (*P* = 0.001), while 740 Y-P increased 1/stem cell frequency in KD-TNC U251 cells (*P* = 0.002) (Table 2).

**Table 2:** ELDA data for stably infected cells after 740 Y-P treatment

Groups	Limiting dilution (wells)			1/Stem cell frequency			*P* value			
	1000	100	10	Lower	Estimate	Upper	KD-NC vs. KD-TNC	KD-NC vs. KD-NC&740 Y-P	KD-TNC vs. KD-TNC&740 Y-P	KD-NC&740 Y-P vs. KD-TNC&740 Y-P
KD-NC	22/24	18/24	8/24	231	138	82.6	0.00179	0.0385	0.00208	0.0337
KD-TNC	19/24	15/24	3/24	520	328	207.0				
KD-NC&740 Y-P	23/24	19/24	12/24	125	79	49.9				
KD-TNC&740 Y-P	22/24	17/24	9/24	234	140	83.7				

*ELDA* extreme limiting dilution analysis

## Discussion

Glioma is one of the most fatal cancers worldwide, with a 5-year survival rate of less than 10% and very limited improvement with treatment. Thus there remains an urgent need to explore novel therapy that may enhance survival in glioma patients (Ostrom et al. 2015). Furthermore, most glioma cases can only be diagnosed at an advanced stage, which is also a major reason for the dismal survival profile of glioma patients. Fortunately, progress has been seen in studies on glioma, among which TNC has shown promise and may play a critical role in glioma pathology. TNC has been revealed to be associated with various aspects of neuron biology, such as the promotion and repression of synapses, neuroinflammation, and neural stem cell and glial progenitor cell proliferation and differentiation (Gottschling et al. 2019; Wiemann et al. 2019; Faissner et al. 2017). In particular, a few findings indicate that TNC might be involved in glioma pathogenesis; however, these findings are far from conclusive. Hence, we conducted the present study and found that (1) TNC promoted glioma cell proliferation and stemness while suppressing glioma cell apoptosis and chemosensitivity to paclitaxel; (2) TNC enhanced PI3K/AKT signaling, and PI3K/AKT signaling activation reversed the effect of TNC on cell function and chemosensitivity to paclitaxel in glioma cells.

Based on a number of former studies, TNC participates in the pathogenesis of glioma via interaction with multiple factors, mostly resulting in tumor progression. As an example, an in vitro experiment found that brain tumor-initiating cell (BTIC) proliferation can be reduced by activated T cells, although this effect is diminished by BTIC secretion of TNC through exosomes (Mirzaei et al. 2018). Another experiment conducted in vivo and in vitro reported that TNC knockdown increased glioma neurosphere cell adhesion and organization of the actin cytoskeleton, and that downregulated TNC expression in the tumor microenvironment was associated with invasion and enhanced proliferation of tumor cells in animal models of glioma (Xia et al. 2016). In addition, an experiment illustrated that interleukin-33 (IL-33) positively regulated TNC expression in glioma cells, and the IL-33/ST2 axis enhanced glioma cell invasion via assembly of TNC (Zhang et al. 2019). Aside from the regulation of cell function, TNC is also involved in the regulation of the angiogenesis of glioma, as reported by a previous study, which revealed that TNC interacts with targets of Yes-associated protein (YAP) to modulate tumor angiogenesis in a bidirectional manner (Rupp et al. 2016). These studies together strongly suggest that TNC serves as a regulator in glioma by mediating the function of glioma cells, BTICs and other corresponding cells. In this study, we found that TNC could enhance the proliferation and stemness of glioma cells while reducing apoptosis and chemosensitivity to paclitaxel. In contrast to previous studies, our study revealed a more comprehensive regulatory role of TNC in glioma, which may derive from the following: First, the capacity of TNC to promote glioma cell malignant activity may reflect its role as an essential regulator of cell adhesion; thus, it may promote glioma cell proliferation but suppress apoptosis by blocking cell adhesion or interacting with other components, such as interleukins, as reported in previous studies (Mirzaei et al. 2018; Xia et al. 2016; Zhang et al. 2019; Rupp et al. 2016). This may also explain the regulatory role of TNC in glioma cell stemness and chemosensitivity, although further functional experiments are needed to confirm this. Second, as revealed in our subsequent experiments, TNC may also modulate glioma cell function and chemosensitivity to paclitaxel through activation of PI3K/AKT signaling.

PI3K/AKT signaling is widely investigated in cancer research as an oncogenic factor, including in studies on glioma. For instance, an in vitro experiment revealed that evodiamine induced glioma cell apoptosis by modulating apoptotic proteins through downregulation of PI3K/AKT signaling and mitogen-activated kinase-like protein (MAPK) phosphorylation (Wang et al. 2018). Another in vitro study reported that in glioma-initiating cells, the repression of PI3K/AKT/mechanistic target of rapamycin (mTOR) signaling and sonic hedgehog signaling markedly reduced cell survival, renewal capacity and tumorigenic potential (Nanta et al. 2019). In addition, this signaling pathway is involved in glioma chemosensitivity. For example, an in vitro study showed that long non-coding RNA DNCAR decreased cell apoptosis caused by cisplatin by inducing the signaling of AXL/PI3K/AKT/necrosis factor-kappa B (NF-κB) in glioma cells (Ma et al. 2018). These findings indicate that PI3K/AKT signaling is a promotor of glioma progression. In the present study, TNC positively regulated PI3K/AKT signaling in glioma cells, and activation of PI3K/AKT signaling reversed the effect of TNC knockdown on cell functions, including proliferation and stemness, and chemosensitivity to paclitaxel. We presume that these results may be related to the fact that PI3K/AKT is deeply involved in many aspects of glioma pathogenesis, and TNC regulation of glioma cell function and chemosensitivity also requires the participation of PI3K/AKT, which is presented as the modulation of PI3K/AKT signaling by TNC in glioma cells (Wang et al. 2018; Nanta et al. 2019; Ma et al. 2018). These findings provide a more profound view of the mechanistic role of TNC in glioma, which may facilitate further investigation of its possible application in the clinical setting.

In summary, TNC acts as an oncogenic factor by promoting cancer cell proliferation and stemness while inhibiting apoptosis and chemosensitivity to paclitaxel in glioma by modulating PI3K/AKT signaling.

## Data Availability

The data sets generated and/or analyzed during the current study are available from the corresponding author on reasonable request.
